# *Scolopsislacrima*, a new species of monocle bream (Teleostei, Perciformes, Nemipteridae) from New Caledonia

**DOI:** 10.3897/zookeys.861.35052

**Published:** 2019-07-08

**Authors:** Jumpei Nakamura, Philippe Béarez, Hiroyuki Motomura

**Affiliations:** 1 Graduate School of Fisheries, Kagoshima University, 4-50-20 Shimoarata, Kagoshima 890-0056, Japan Kagoshima University Kagoshima Japan; 2 UMR 7209 AASPE, Muséum national d’Histoire naturelle, 55 rue Buffon, 75005 Paris, France Muséum national d’Histoire naturelle Paris France; 3 The Kagoshima University Museum, 1-21-30 Korimoto, Kagoshima 890-0065, Japan The Kagoshima University Museum Kagoshima Japan

**Keywords:** Grande-Terre Island, morphology, *
Scolopsis meridiana
*, taxonomy

## Abstract

The new monocle bream *Scolopsislacrima***sp. nov.** is described from a single specimen (213.6 mm standard length) collected from Grande-Terre Island, New Caledonia. The new species closely resembles *S.meridiana*, both species having the upper part of the pectoral-fin base with reddish blotch when fresh, two bands across the top of the snout, a dorsal scaled area on the head reaching anteriorly to between the anterior margin of the eye and anterior nostril, a similar number of lateral-line scales, and absence of a small antrorse spine below the eye. However, *S.lacrima***sp. nov.** is distinguished from *S.meridiana* by having diagonal lines on the body absent (vs. 18–20 diagonal lines in the latter), a dark longitudinal band below the lateral line (vs. longitudinal lines absent), the caudal fin central area not patterned (vs. with several dark horizontal lines), a narrower body and shallower caudal peduncle.

## Introduction

The monocle bream genus *Scolopsis* Cuvier, 1814 is widespread throughout shallow Indo-West Pacific tropical and subtropical waters, some species being marketed in Southeast Asia (Russell 1990, 2001). The genus was reviewed by Russell (1990), who recognised 16 valid species; it was characterised by a distinct posteriorly-directed suborbital spine, the suborbital area without scales, the posterior margin of the preopercle coarsely denticulate or serrate, and jaws without canine teeth. Subsequently, *Scolopsisigcarensis* Mishra, Biswas, Russell, Satpathy & Selvanayagam, 2013 and *Scolopsismeridiana* Nakamura, Russell, Moore & Motomura, 2018 were described, and *Scolopsistorquata* (Cuvier, 1830) recognised as a valid species by Psomadakis et al. (2015). Accordingly, 19 valid species are currently recognised in the genus (Russell 1990, Mishra et al. 2013, Psomadakis et al. 2015, Nakamura et al. 2018).

During a taxonomic study of *Scolopsis*, a single specimen from New Caledonia, having a distinctively elongate body and unique colouration, was examined. It is described herein as a new species of *Scolopsis*.

## Materials and methods

Counts and proportional measurements followed Nakamura et al. (2018). All measurements were made with calipers to the nearest 0.1 mm. Standard length is abbreviated as SL. Institutional codes follow Sabaj (2016), with the following addition: Département d’Archéologie du Service des Musées de Nouméa, New Caledonia (**DASMN**). Examined specimens of *S.meridiana* and *Scolopsistaenioptera* (Cuvier, 1830) are listed in Nakamura et al. (2018).

### Scolopsis lacrima sp. nov.

Taxon classificationAnimaliaSpariformesNemipteridae

http://zoobank.org/5AF65765-E484-41A9-ABB2-E88A93034AE5

**Figure 1.**
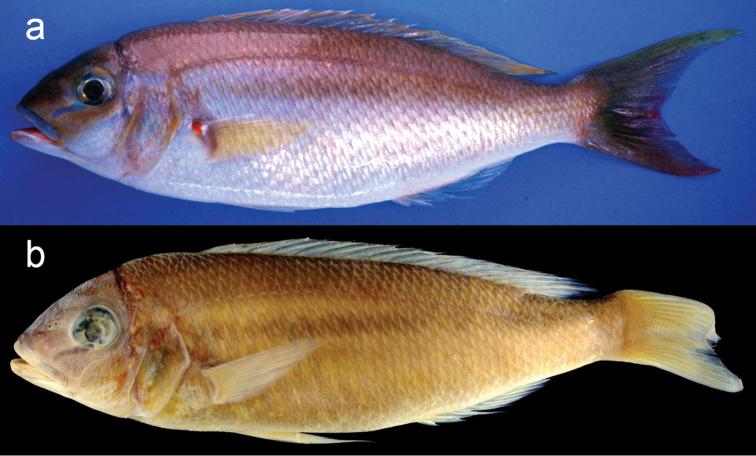
Holotype of *Scolopsislacrima* sp. nov., MNHN 2002-2930, 213.6 mm SL, Grande-Terre Island, New Caledonia **a** fresh condition (photo by P. Béarez) **b** preserved condition.

**Figure 2.**
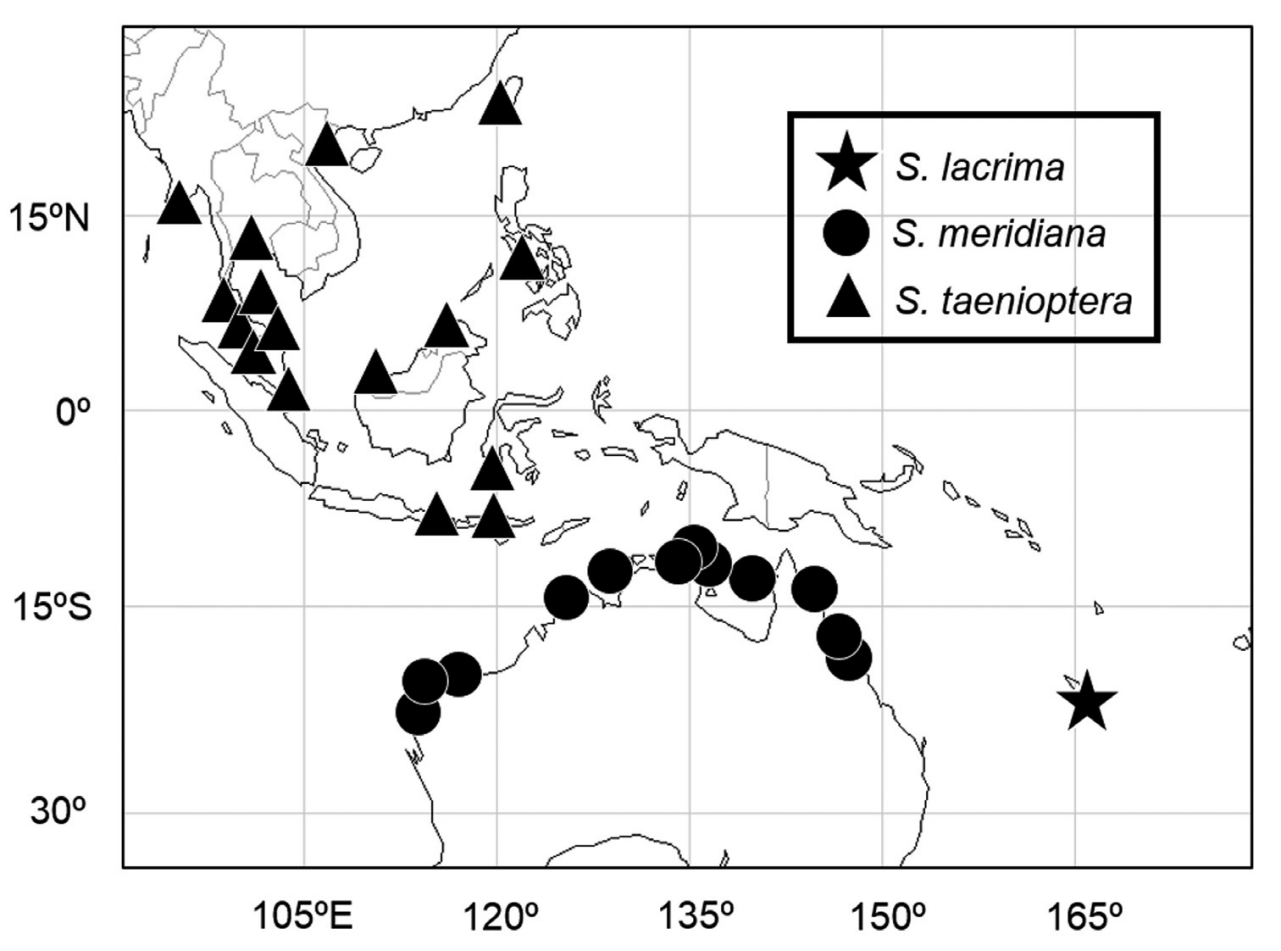
Distribution records of *Scolopsislacrima* sp. nov. (star), *S.meridiana* (circles), and *S.taenioptera* (triangles), based on specimens examined in Nakamura et al. (2018) and this study.

**Figure 3.**
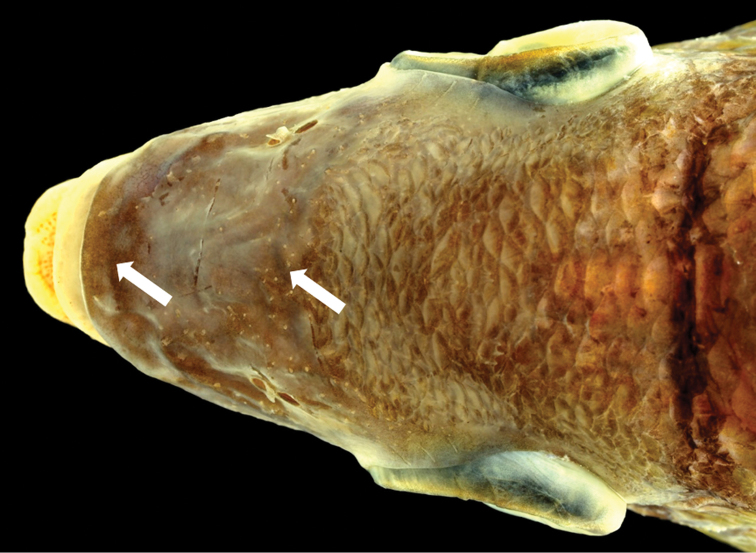
Dorsal view of snout of *Scolopsislacrima* sp. nov. (MNHN 2002-2930, holotype, 213.6 mm SL).

Figures 1 , 2 , 3 , 5a , 6 , 7 New English name: Teary Monocle Bream

Scolopsis taeniopterus (non Cuvier): Béarez 2003: 62, fig. 1 (Nouméa, Grande-Terre Island, New Caledonia).
Scolopsis taenioptera (non Cuvier): Fricke et al. 2011: 401 (New Caledonia).

#### Holotype.

MNHN 2002–2930, 213.6 mm SL, Nouméa, Grande-Terre Island, New Caledonia, 1 Aug 2002, purchased at market by P. Béarez.

#### Diagnosis.

A species of *Scolopsis* with the following combination of characters: pectoral-fin rays 17; lateral-line scales 47; no antrorse spine below eye; dorsal scaled area on head reaching anteriorly to between anterior margin of eye and anterior nostril; bony opercular ridge and lower limb of preopercle without scales; 3^rd^ anal-fin spine longer than 2^nd^ anal-fin spine; narrow body, its depth at dorsal, pelvic, and anal fin origins 29.2, 29.5 and 26.6% of SL, respectively; caudal-peduncle depth 10.4% of SL; head length 29.9% of SL; upper part of pectoral-fin base with reddish blotch when fresh; two dark bands across dorsum of snout; body below lateral line with a dark longitudinal band, without diagonal lines; no blotches or lines on central area of caudal fin.

#### Description.

Dorsal-fin rays X, 9; anal-fin rays III, 7; pectoral-fin rays (left / right) 17 / 17; pored lateral-line scales 47; pelvic-fin rays I, 5; scale rows above lateral line 5; scale rows below lateral line 10; gill rakers (upper / lower) 5 / 8; preopercle scale rows (behind eye) 3; preopercle scale rows (below eye) 4. The following morphometrics are expressed as percentages of SL: body depth at dorsal-fin origin 29.2; body depth at pelvic-fin origin 29.5; body depth at anal-fin origin 26.6; body depth at posterior margin of orbit 23.7; body depth at anterior margin of orbit 17.5; pre-dorsal-fin length 32.7; pre-pelvic-fin length 37.9; pectoral-pelvic length 15.4; pre-anus length 61.4; head length 29.9; snout length 11.4; posterior nostril (horizontal) 0.8; posterior nostril (vertical) 0.8; upper-jaw length 10.7; orbit diameter 7.8; interorbital width 10.0; suborbital depth 5.7; caudal-peduncle length 22.8; caudal-peduncle depth 10.4; dorsal-fin base length 54.1; 1^st^ dorsal-fin spine length 6.2; 2^nd^ dorsal-fin spine length 8.5; 3^rd^ dorsal-fin spine length 10.4; 4^th^ dorsal-fin spine length 10.8; 5^th^ dorsal-fin spine length 11.0; 6^th^ dorsal-fin spine length 11.0; 7^th^ dorsal-fin spine length 10.9; 8^th^ dorsal-fin spine length 11.0; 9^th^ dorsal-fin spine length 10.7; 10^th^ dorsal-fin spine length 10.4; longest dorsal-fin soft ray length 16.0; 1^st^ anal-fin spine length 3.9; 2^nd^ anal-fin spine length 7.4; 3^rd^ anal-fin spine length 8.0; anal-fin base length 15.0; pectoral-fin length 21.3; pelvic-fin spine length (measured on right side because the left side damaged) 13.8; longest pelvic-fin soft ray length 24.0.

Body oblong, rather compressed, deepest at pelvic-fin origin. Dorsal profile rising from snout tip to dorsal-fin origin, lowering slightly between origins of 1^st^ to 10^th^ dorsal-fin spines, thereafter more steeply to caudal peduncle. Ventral profile of body lowering from lower-jaw tip to anus, thereafter rising to caudal peduncle. Dorsal-fin origin just above posteriormost point of opercle, base extending posterior to posteriormost point of anal-fin base. First to 5^th^ dorsal-fin spines gradually lengthening, 5^th^ to 8^th^ spine lengths similar, 8^th^ to 10^th^ spines gradually shortening. Seventh dorsal-fin soft ray longest. All dorsal-fin soft rays non-filamentous. Uppermost point of pectoral-fin base slightly posterior to posteriormost point of opercle. Lowermost point of pectoral-fin base anterior to pelvic-fin origin. Posterior tip of pectoral fin pointed, reaching to vertical through 7^th^ dorsal-fin spine origin. Pelvic-fin origin posterior to dorsal-fin origin. Posterior tip of depressed pelvic fin reaching anus, not reaching anal-fin origin. Anal-fin origin below 1^st^ dorsal-fin ray origin, ending below 6^th^ dorsal-fin ray origin. First anal-fin spine shortest, 3^rd^ spine longest. Caudal-fin forked, upper lobe longer than lower lobe. Posterior tip of both lobes of caudal fin pointed, non-filamentous. Anus oblong, anterior to anal-fin origin. Eye and pupil round. Lower margin of eye above a line from snout tip to uppermost part of pectoral-fin base. Nostrils round, paired, positioned close together anterior to orbit, anterior nostril with small dermal flap. Snout pointed. Posterior tip of maxilla not reaching to vertical through anterior margin of eye. Distinct suborbital spine posteriorly directed. Small antrorse spine below eye absent. Posterior margins of suborbital and preopercle serrated. Scales ctenoid; both lips, snout, area around eye, and bony opercular ridge and lower limb of preopercle scaleless. Lateral line complete, originating above opercle, extending to central part of caudal-fin base. Both jaws with small conical teeth, forming dense bands. Canine teeth absent. Gill rakers long, slender.

#### Colour when fresh.

Based on colour photograph of holotype (MNHN 2002–2930; Fig. 1a). Head and body reddish-brown dorsally, silver-white ventrally. Upper lip blue. Two brown bands across dorsum of snout, connecting eyes. Upper band above posterior nostril, lower band below anterior nostril. Blue band on suborbital from anteroventral margin of orbit to just short of upper lip. Gill membrane yellow. A dark longitudinal band below lateral line from behind posterior margin of opercle to caudal peduncle. No diagonal lines on body. Distinct reddish blotch on upper end of pectoral-fin base. Pectoral fin pale yellow. Dorsal-fin membrane yellowish, semi-transparent, with yellow outer margin. Pelvic and anal fins white. Several indistinct yellowish longitudinal stripes on caudal peduncle. Upper base of caudal fin with blue blotch. Caudal fin red with yellowish upper margin. Central area of caudal fin without blotches or lines.

#### Colour in alcohol.

(Fig. 1b) Head, body, and caudal fin uniformly pale brown. Three dark bands radiating from orbit. A dark brown longitudinal band below lateral-line. Dorsal, pectoral, pelvic, and anal fins pale yellow.

#### Distribution.

Currently known only from New Caledonia (Fig. 2).

#### Etymology.

The specific name *lacrima* is derived from Latin meaning a tear, in reference to the distinct blue band below the eye of the species.

#### Remarks.

**Figure 4.**
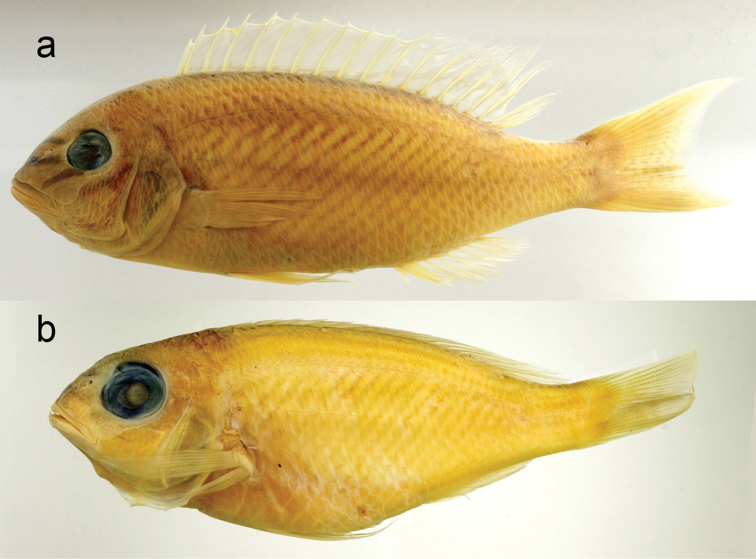
*Scolopsismeridiana***a** CSIRO H 4029–01, holotype, 194.8 mm SL, Western Australia, Australia **b** AMS I.21957-013, paratype, 94.9 mm SL, Northern Territory, Australia.

**Figure 5.**
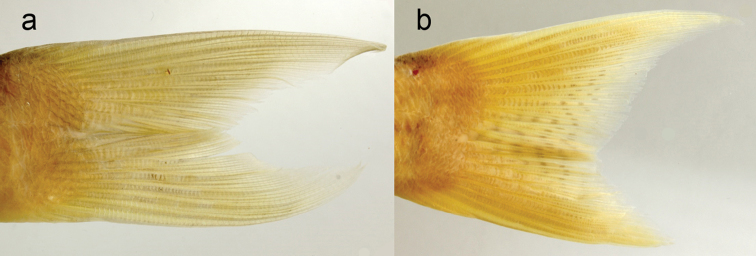
Caudal fin **a***Scolopsislacrima* sp. nov. (MNHN 2002-2930, holotype, 213.6 mm SL, flip horizontal) **b***S.meridiana* (CSIRO H 4029–01, holotype, 194.8 mm SL).

The new species is assignable to the genus *Scolopsis*, defined by Russell (1990, 2001), due to its distinct posteriorly-directed suborbital spine, the suborbital area without scales, the posterior margin of the preopercle coarsely denticulate or serrate, and jaws without canine teeth. *Scolopsislacrima* (Fig. 1) is similar to *S.meridiana* (Fig. 4) and *S.taenioptera*, the three species uniquely sharing the upper pectoral-fin base with a reddish blotch when fresh (Fig. 1a) (vs. reddish blotch absent in all other congeners). *Scolopsislacrima* and *S.meridiana* are easily distinguished from *S.taenioptera* by having two bands across the top of the snout (Fig. 3) [vs. single band; Nakamura et al. (2018: fig. 4)]; detailed comparisons of *S.meridiana* with *S.taenioptera* were given in Nakamura et al. (2018). *Scolopsislacrima* is distinguished from *S.meridiana* by the lack of diagonal lines on the body below the lateral line (Fig. 1) [vs. 18–20 brown diagonal lines in preserved specimens in *S.meridiana* (Fig. 4)], presence of a dark longitudinal band on the body below the lateral line (Fig. 1) [vs. longitudinal band absent (Fig. 4a), although young individuals (< 108.9 mm SL) may rarely have an indistinct dark longitudinal band (Fig. 4b)], and lack of blotches centrally on the caudal fin (Fig. 5a) [vs. several small poorly-defined blotches (Fig. 5b), although young individuals (< 108.9 mm SL) may rarely lack blotches (Fig. 4b)]. Moreover, body depths at the origins of the dorsal (29.2% of SL vs. 30.8–35.0% in *S.meridiana*, Fig. 7a), pelvic (29.5% vs. 31.6–38.4%, Fig. 7b), and anal fins (29.6% vs. 28.1–32.5%, Fig. 7c) are narrower in *S.lacrima*, and the caudal-peduncle depth (10.4% of SL vs. 11.4–13.1%, Fig. 7d) and head length (29.9% vs. 30.1–32.7%) less.

**Figure 6.**
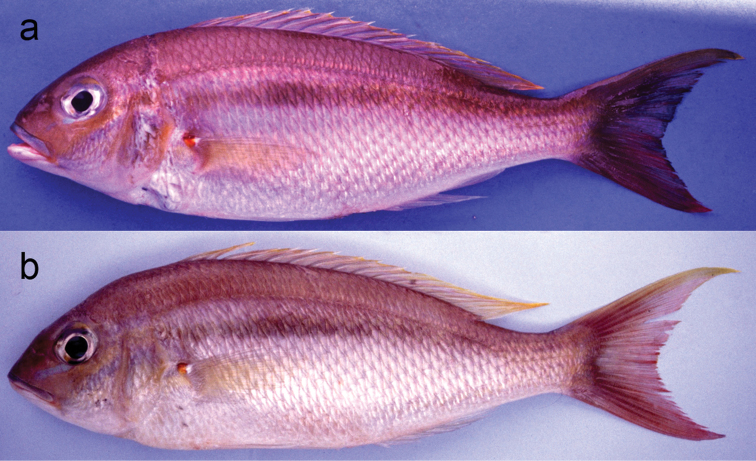
Colour photographs of *Scolopsislacrima* sp. nov., Grande-Terre Island, New Caledonia (photos by P. Béarez) **a**DASMN-52, 187 mm SL **b** MNHN-ICOS-00437, 192 mm SL.

**Figure 7.**
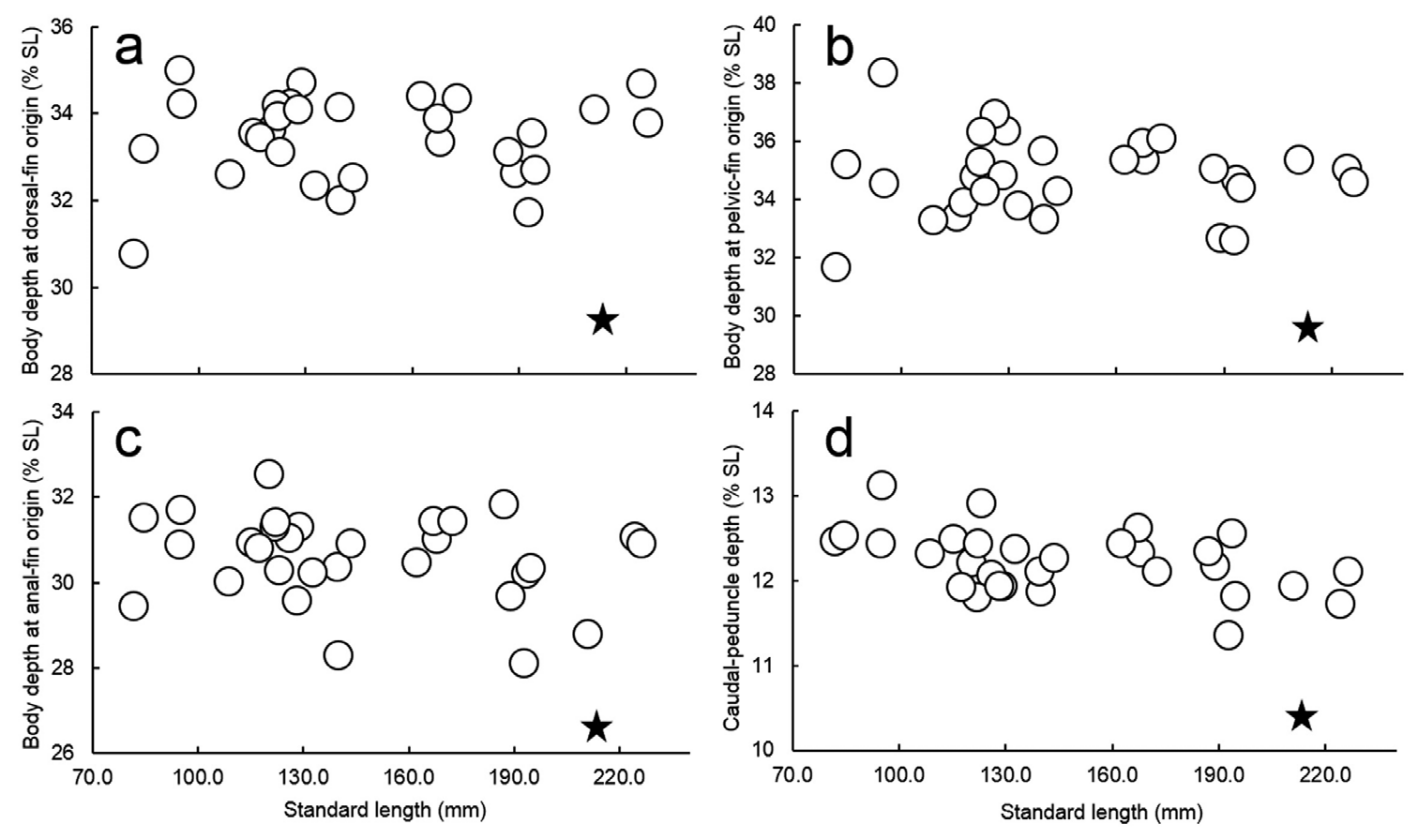
Relationships of (**a**) body depth at dorsal-fin origin, (**b**) body depth at pelvic-fin origin, (**c**) body depth at anal-fin origin, and (**d**) caudal-peduncle depth % of SL to SL in *Scolopsislacrima* sp. nov. (diamond) and *S.meridiana* (circles).

Béarez (2003) reported three specimens [MNHN 2002–2930 (designated here as the holotype of *S.lacrima*; Fig. 1), MNHN-ICOS-00437, and DASMN-52] as *Scolopsistaeniopterus* (Cuvier, 1830) from New Caledonia. The latter two specimens have been reduced to bones and otoliths only. However, fresh colour photographs of both specimens prior to dissection (Fig. 6) support their identification here as *S.lacrima*.

*Scolopsismeridiana* and *S.taenioptera* are restricted to northern Australia and Southeast Asia, respectively (Fig. 2), probably not occurring in New Caledonia, suggesting that the three species of *Scolopsis* with a reddish blotch on the upper part of the pectoral-fin base are allopatrically distributed in the Indo-West Pacific.

## Supplementary Material

XML Treatment for Scolopsis lacrima
